# Percutaneous coronary intervention vs. coronary artery bypass grafting in emergency and non-emergency unprotected left-main revascularization

**DOI:** 10.1186/s40001-023-01189-1

**Published:** 2023-07-01

**Authors:** Amin Daoulah, Abdulrahman H. Alqahtani, Ahmed Elmahrouk, Nooraldaem Yousif, Wael Almahmeed, Amr A. Arafat, Turki Al Garni, Mohammed A. Qutub, Ziad Dahdouh, Mohammed Alshehri, Ahmad S. Hersi, Majed M. Malak, Syifa R. Djunaedi, Ayesha Zaidi, Maryam Jameel Naser, Wael Qenawi, Abdelmaksoud Elganady, Taher Hassan, Vincent Ball, Youssef Elmahrouk, Adnan Fathey Hussien, Badr Alzahrani, Reda Abuelatta, Ehab Selim, Ahmed Jamjoom, Khalid Z. Alshali, Shahrukh Hashmani, Wael Refaat, Hameedullah M. Kazim, Mohamed Ajaz Ghani, Haitham Amin, Ahmed M. Ibrahim, Abdulwali Abohasan, Mohamed N. Alama, Mohammed Balghith, Ibrahim A. M. Abdulhabeeb, Osama Ahmad, Mohamed Ramadan, Ahmed A. Ghonim, Abeer M. Shawky, Husam A. Noor, Abdulrahman M. Alqahtani, Faisal Al Samadi, Seraj Abualnaja, Rasha Taha Baqais, Abdulkarim Alhassoun, Issam Altnji, Mushira Khan, Abdulaziz Alasmari, Alwaleed Aljohar, Niranjan Hiremath, Jairam Aithal, Amir Lotfi

**Affiliations:** 1 Department of Cardiovascular Medicine, King Faisal Specialist Hospital & Research Center, P.O. Box: 40047, Jeddah, 21499 Kingdom of Saudi Arabia; 2 Department of Emergency Medicine, King Abdulaziz Medical City, Riyadh, Kingdom of Saudi Arabia; 3 Department of Cardiothoracic Surgery, Faculty of Medicine, Tanta University, Tanta, Egypt; 4 Department of Cardiology, Mohammed Bin Khalifa Specialist Cardiac Center, Awali, Kingdom of Bahrain; 5 Heart & Vascular Institute, Cleveland Clinic Abu Dhabi, Abu Dhabi, UAE; 6 Department of Cardiology, Prince Sultan Cardiac Center, Riyadh, Kingdom of Saudi Arabia; 7 Cardiology Center of Excellence, Department of Medicine, King Abdulaziz University, Jeddah, Kingdom of Saudi Arabia; 8 Department of Cardiovascular Medicine, King Faisal Specialist Hospital & Research Center, Riyadh, Kingdom of Saudi Arabia; 9 Department of Cardiology, Prince Khaled Bin Sultan Cardiac Center, Khamis Mushait, Kingdom of Saudi Arabia; 10 Department of Cardiac Sciences, King Fahad Cardiac Center, King Saud University, Riyadh, Kingdom of Saudi Arabia; 11 Department of Medicine, King Abdulaziz University, Rabigh, Kingdom of Saudi Arabia; 12 Department of Medicine, University of Massachusetts Chan Medical School - Baystate Medical Center, Springfield, MA 01199 USA; 13 Department of Medicine, Baystate Medical Center, 759 Chestnut St, Springfield, MA USA; 14 Department of Cardiology, Dr. Erfan and Bagedo General Hospital, Jeddah, Kingdom of Saudi Arabia; 15 Faculty of Medicine, Al-Azhar University, Cairo, Egypt; 16 Department of Cardiology, Bugshan General Hospital, Jeddah, Kingdom of Saudi Arabia; 17 Department of Emergency Medicine, Cleveland Clinic, Cleveland, OH USA; 18 Faculty of Medicine, Tanta University, Tanta, Egypt; 19 Department of Cardiology, International Medical Center, Jeddah, Kingdom of Saudi Arabia; 20 Department of Cardiology, Madinah Cardiac Center, Madinah, Kingdom of Saudi Arabia; 21 Department of Cardiology, Alhada Armed Forces Hospital, Taif, Kingdom of Saudi Arabia; 22 Department of Medicine, Faculty of Medicine, King Abdulaziz University, Jeddah, Kingdom of Saudi Arabia; 23 Department of Cardiology, Prince Sultan Cardiac Center, Al Hassa, Kingdom of Saudi Arabia; 24 Department of Cardiology, Saudi German Hospital, Jeddah, Kingdom of Saudi Arabia; 25 Department of Cardiology, Prince Sultan Cardiac Center, Qassim, Kingdom of Saudi Arabia; 26 King Abdulaziz Cardiac Center, College of Medicine, King Saud Bin Abdulaziz University for Health Science, Riyadh, Kingdom of Saudi Arabia; 27 Department of Cardiology, King Abdulaziz Specialist Hospital, Al Jawf, Kingdom of Saudi Arabia; 28 Department of Cardiology, King Fahad Medical City, King Salman Heart Center, Riyadh, Kingdom of Saudi Arabia; 29 Department of Cardiac Surgery, King Fahd Armed Forces Hospital, Jeddah, Kingdom of Saudi Arabia; 30 Department of Anesthesia, King Faisal Specialist Hospital & Research Center, Jeddah, Kingdom of Saudi Arabia; 31 Department of Cardiology, Our Lady of Lourdes Hospital, Drogheda, Ireland; 32 Al Faisal University, Riyadh, Kingdom of Saudi Arabia; 33 Department of Cardiology, Yas Clinic, Khalifa City A, UAE; 34 Department of Cardiovascular Medicine, University of Massachusetts Chan Medical School - Baystate Medical Center, Springfield, MA 01199 USA

**Keywords:** Emergency PCI, Emergency CABG, ULMCA, Outcomes, Gulf

## Abstract

**Background:**

The optimal revascularization strategy in patients with left main coronary artery (LMCA) disease in the emergency setting is still controversial. Thus, we aimed to compare the outcomes of percutaneous coronary interventions (PCI) vs. coronary artery bypass grafting (CABG) in patients with and without emergent LMCA disease.

**Methods:**

This retrospective cohort study included 2138 patients recruited from 14 centers between 2015 and 2019. We compared patients with emergent LMCA revascularization who underwent PCI (*n* = 264) to patients who underwent CABG (*n* = 196) and patients with non-emergent LMCA revascularization with PCI (*n* = 958) to those who underwent CABG (*n* = 720). The study outcomes were in-hospital and follow-up all-cause mortality and major adverse cardiovascular and cerebrovascular events (MACCE).

**Results:**

Emergency PCI patients were older and had a significantly higher prevalence of chronic kidney disease, lower ejection fraction, and higher EuroSCORE than CABG patients. CABG patients had significantly higher SYNTAX scores, multivessel disease, and ostial lesions. In patients presenting with arrest, PCI had significantly lower MACCE (*P* = 0.017) and in-hospital mortality (*P* = 0.016) than CABG. In non-emergent revascularization, PCI was associated with lower MACCE in patients with low (*P* = 0.015) and intermediate (*P* < 0.001) EuroSCORE. PCI was associated with lower MACCE in patients with low (*P* = 0.002) and intermediate (*P* = 0.008) SYNTAX scores. In non-emergent revascularization, PCI was associated with reduced hospital mortality in patients with intermediate (*P* = 0.001) and high (*P* = 0.002) EuroSCORE compared to CABG. PCI was associated with lower hospital mortality in patients with low (*P* = 0.031) and intermediate (*P* = 0.001) SYNTAX scores. At a median follow-up time of 20 months (IQR: 10–37), emergency PCI had lower MACCE compared to CABG [HR: 0.30 (95% CI 0.14–0.66), *P* < 0.003], with no significant difference in all-cause mortality between emergency PCI and CABG [HR: 1.18 (95% CI 0.23–6.08), *P* = 0.845].

**Conclusions:**

PCI could be advantageous over CABG in revascularizing LMCA disease in emergencies. PCI could be preferred for revascularization of non-emergent LMCA in patients with intermediate EuroSCORE and low and intermediate SYNTAX scores.

## Background

Left main coronary artery (LMCA) disease represents a highly morbid condition with a poor prognosis if not revascularized [1]. Furthermore, infarction related to LMCA disease is associated with a high myocardial jeopardy score and extensive ischemia to multiple large coronary territories. It, therefore, carries an increased risk of complications, including left ventricular (LV) systolic dysfunction, cardiogenic shock, and death [2]. Historically, revascularization with coronary artery bypass grafting (CABG) has been the gold standard for LMCA disease in stable coronary artery disease (CAD). With current stent technology and guideline-based acute coronary syndrome (ACS) management, percutaneous coronary intervention (PCI) revascularization for the left-main disease is now considered non-inferior to CABG in patients with stable CAD and low to intermediate SYNTAX scores [2, 3]. Published reports indicated that patients undergoing PCI revascularization for unprotected LMCA disease are increasing, and CABG procedures are decreasing [4].

The number of patients with LMCA disease and high anatomical complexity included in randomized controlled trials is low because they are usually excluded [5]. Consequently, the risk estimates and confidence intervals are imprecise; however, they suggest a trend toward better survival with CABG [3]. There is a paucity of data to guide left-main revascularization in emergency presentations, including cardiac arrest and cardiogenic shock complicating acute myocardial infarction. The optimal revascularization strategies in emergent and non-emergent LMCA revascularization have not been thoroughly evaluated. Considering this gap in the evidence, we conducted this study to analyze the outcomes of reperfusion strategies (PCI versus CABG) for emergent and non-emergent LMCA disease.

## Methods

### Study design, patient population

The Gulf left-main registry contains data about LMCA revascularization with either PCI or CABG from 14 cardiac centers in the Gulf Area [6, 7]. Patients were recruited from January 2015 to December 2019. A total of 2657 patients with significant LMCA disease were identified. The registry included 2138 patients with unprotected left-main coronary artery (ULMCA) disease; 460 had emergent, and 1678 had non-emergent revascularization. We compared patients with emergent LMCA disease who had PCI (*n* = 264) to CABG patients (*n* = 196) and patients with non-emergent LMCA who had PCI (*n* = 958) to those who had CABG (*n* = 720). We excluded patients with previous LMCA PCI (*n* = 37), Unprotected LMCA treated medically (*n* = 193), concomitant valvular or aortic surgery (*n* = 115), and those with protected LMCA disease with patent grafts (*n* = 174).

### Definitions

The emergent LMCA revascularization group included all ST-elevation myocardial infarction (STEMI) cases and high-risk non-ST-elevation acute coronary syndrome (NSTE-ACS). High-risk NSTE-ACS included patients in cardiac arrest and/or cardiogenic shock. The non-emergent LMCA revascularization group included all remaining patients who underwent LMCA revascularization.

Significant LMCA disease was defined as luminal stenosis greater than 50%.

Unprotected LMCA disease was defined as LMCA disease without previous bypass grafts to either the left anterior descending (LAD) or circumflex coronary artery.

Preprocedural patient risk stratification was performed using the European System for Cardiac Operative Risk Evaluation (EuroSCORE II) [8]. The EuroSCORE II is divided into three categories [low score (0–2), medium (3–5) and high (≥ 6)].

The SYNTAX score was used to score the angiographic lesions [9]. The SYNTAX score was divided into three groups [low score (≤ 22), intermediate (23–32), and high (> 33)].

Medina classification was used for LM bifurcation lesions [10].

Bleeding events were defined according to the International Society of Thrombosis and Hemostasis (ISTH) Scientific and Standardization Committee's (SSC) statement [11, 12].

### Clinical assessment and clinical follow-up

The data pertaining to the patient's demographics, presentation, medications at the time of discharge, and in-patient and follow-up outcomes were analyzed in both emergent and non-emergent LMCA revascularization procedures. Clinical outcomes included hospital and follow-up all-cause mortality and major adverse cardiac and cerebrovascular events (MACCEs). MACCEs included the composite endpoint of myocardial infarction (MI), cerebrovascular events, target lesion revascularization, target vessel revascularization, and cardiac or noncardiac mortality. The mechanism by which follow-up events were recorded was based either on ICD coding diagnosis or on clinical description based on clinical diagnosis by admitting physicians in the electronic health record (EHR).

### Ethical approval

This study was approved by the Institutional Review Board of King Faisal Specialist Hospital and Research Center in Riyadh (12-November 2020—RAC # 2201226: Gulf-LM Registry) and was carried out per local guidelines and ethical guidelines of the Declaration of Helsinki [13]. The IRB waived informed consent for this study due to its retrospective and observational nature and the absence of any patient-identifying information.

### Statistical analysis

Stata 17 (Stata Corp- College Station- TX- USA) was used for all analyses. Quantitative data were evaluated for normality distribution using the Shapiro‒Wilk test, and normally distributed variables were compared using the Student t-test and presented as the mean and standard deviation. Skewed quantitative data were expressed as the median (interquartile range) and compared with the Wilcoxon test. Qualitative data were expressed as numbers and percentages and compared with the Chi-squared or Fisher's exact test if the expected frequency was less than five. The distribution of time-to-event outcomes (MACCE and survival) was plotted using Kaplan‒Meier curves and compared with the log-rank test. Multivariable logistic regression analysis was used to evaluate factors associated with hospital MACCE and mortality in both emergent and non-emergent LMCA revascularization separately. Data were grouped into four models; baseline data, presentations, EuroSCORE, and SYNTAX score. Univariable logistic regression analysis was performed, and all variables were included in a stepwise forward selection regression model. Significant variables were retained in the final model. The interaction between PCI/CABG and presentation, EuroSCORE, or SYNTAX score was evaluated. The odds ratio and its 95% confidence interval, and P-values were reported. Collinearity was tested with the variance inflation factor (VIF), and all variables included in the model had VIF < 1.5. Multivariable Cox regression analysis was used to evaluate factors associated with the time-to-event outcomes (MACCE and survival). Stepwise forward selection was used, and a *P* < 0.05 was used to retain the variables in the final model. The model contained all patients with emergent and non-emergent LMCA revascularization, and the interaction between emergent/non-emergent revascularization and the technique (PCI vs. CABG) was evaluated in the final model. All preoperative and angiographic variables were included. The hazard ratio and its 95% confidence interval and P-value were reported. A P-value of less than 0.05 was considered statistically significant.

## Results

### Preprocedural and procedural technique and lesion characteristics

In the emergent LMCA revascularization group, patients who underwent PCI were older and had a significantly higher prevalence of chronic kidney disease than those who underwent CABG. Peripheral arterial disease was more common in patients with non-emergent PCI than non-emergent CABG. The EuroSCORE II was significantly higher in patients with PCI than those with CABG (Table 1).

**Table 1:** Comparison of the baseline demographics between patients who had emergency left main (LM) and non-emergency LM revascularization

	Overall, *n* = 2138	Emergent LM revascularization *n* = 460 (21.5%)		*P*-Value	Non-emergent LM revascularization *n* = 1678 (78.5%)		*P* Value
		PCI (*n* = 264)	CABG (*n* = 196)		PCI (*n* = 958)	CABG (*n* = 720)	
Baseline characteristics							
Age (years), median (IQR)	64 (57–70)	63 (56–72)	60 (54- 68)	0.004	66 (60–72)	62 (55–69)	< 0.001
Body mass index (kg/m^2^), median (IQR)	28 (25- 32)	28 (25–31)	28 (25–30)	0.269	29 (26–33)	28 (25–31)	< 0.001
Gender (male), *n* (%)	1678 (78.5%)	202 (76.52%)	170 (86.73%)	0.006	692 (7223%)	614 (85.28%)	< 0.001
Smoker, *n* (%)	849 (39.7%)	85 (32.20%)	83 (42.35%)	0.025	394 (41.13%)	287 (39.86%)	0.601
Diabetes mellitus, *n* (%)	1468 (68.7%)	185 (70.08%)	139 (70.92%)	0.845	619 (64.61%)	525 (72.92%)	< 0.001
Hypertension, *n* (%)	1495 (70.4%)	173 (65.78%)	122 (62.24%)	0.434	686 (72.67%)	514 (71.39%)	0.564
Dyslipidemia, *n* (%)	1463 (69.0%)	179 (68.06%)	125 (63.78%)	0.337	642 (68.15%)	517 (71.81%)	0.108
Congestive heart failure, *n* (%)	261 (12.2%)	44 (16.67%)	23 (11.73%)	0.138	152 (15.87%)	42 (5.83%)	< 0.001
Peripheral arterial disease, *n* (%)	246 (11.5%)	28 (10.61%)	20 (10.20%)	0.889	156 (16.28%)	42 (5.83%)	< 0.001
Cerebral vascular accident, *n* (%)	222 (10.4%)	30 (11.36%)	11 (5.61%)	0.032	149 (15.55%)	32 (4.44%)	< 0.001
Chronic kidney disease, *n* (%)	437 (20.4%)	62 (23.48%)	29 (14.80%)	0.021	254 (26.51%)	92 (12.78%)	< 0.001
Atrial fibrillation, *n* (%)	183 (8.6%)	27 (10.23%)	12 (6.12%)	0.118	127 (13.26%)	17 (2.36%)	< 0.001
Previous myocardial infarction, *n* (%)	613 (28.7%)	84 (31.82%)	60 (30.61%)	0.783	334 (34.86%)	135 (18.75%)	< 0.001
EuroSCORE, median (IQR)	2.9 (1.26–4.56)	3.9 (2.01–7)	2.45 (1.27–5)	< 0.001	1.75 (1.04–3.65)	3.65 (1.6–4.65)	< 0.001

CABG: coronary artery bypass graft surgery, PCI: percutaneous coronary intervention, EuroSCORE score: European system for cardiac operative risk evaluation, IQR: interquartile range

Eighteen patients (1%) presented with cardiac arrest (50% ventricular fibrillation), 82 (3.8%) with cardiogenic shock, and 52 (2.4%) with both. Ninety-five percent of cardiac arrests occurred in-hospital, and 95% of cardiogenic shocks occurred pre-revascularization. More patients in cardiogenic shock and cardiac arrest had PCI. Three hundred eighty-nine patients (84.6%) presented with STEMI; 81 (21%) were in cardiogenic shock and cardiac arrest. Seventy-one (15.4%) patients presented with high-risk (arrest and shock) non-ST-elevation acute coronary syndrome (NSTE-ACS). While more patients with STEMI underwent CABG, more patients presenting with NSTE-ACS underwent PCI. Patients who underwent PCI had significantly lower left ventricular ejection fractions than those who underwent CABG (Table 2). The most frequently observed anatomical pattern was Medina 1,1,1 lesion (35.5%), followed by 1,1,0 (18.8%). Patients who underwent CABG had significantly higher SYNTAX scores, multivessel disease, and ostial lesions. However, PCI patients had significantly lower SYNTAX scores and more isolated LMCA disease or LMCA with either single or double-vessel disease and distal bifurcation lesions (Table 3).

**Table 2:** Comparison of the hospital presentation between patients who had emergency left main (LM) vs. non-emergency LM revascularization

	Overall, *n* = 2138	Emergent LM revascularization *n* = 460 (21.5%)		P-Value	Non-emergent LM revascularization *n* = 1678 (78.5%)		*P* Value
		PCI (*n* = 264)	CABG (*n* = 196)		PCI (*n* = 958)	CABG (*n* = 720)	
Hospital presentation							
Cardiac arrest, *n* (%)	18 (1%)	7 (2.65%)	11 (5.61%)	0.105	NA	NA	NA
Types, *n* (%)							
Ventricular fibrillation	9 (50%)	2 (28.57%)	7 (63.64%)	0.335	NA	NA	NA
Pulseless electrical activity	6 (33.3%)	3 (42.86%)	3 (27.27%)	0.627	NA	NA	NA
Asystole	3 (16%)	2 (28.57%)	1 (9.09%)	0.528	NA	NA	NA
Location, *n* (%)							
Out of hospital cardiac arrest (OHCA)	1 (5.5%)	1 (14.29%)	0	0.389	NA	NA	NA
In-hospital cardiac arrest (IHCA)	17 (94.5%)	6 (85.71%)	11 (100%)	0.389	NA	NA	NA
Timing, *n* (%)							
Pre coronary angiography	18 (100%)	7 (100%)	11 (100%)	> 0.99	NA	NA	NA
Post coronary angiography	0 (0%)	0	0	> 0.99	NA	NA	NA
Cardiogenic shock and cardiac arrest, *n* (%)	52 (2.4%)	44 (16.67%)	8 (4.08%)	< 0.001			
Timing, *n* (%)							
Pre coronary angiography	47 (90.4%)	43 (97.73%)	4 (50%)	0.001	NA	NA	NA
Post coronary angiography	5 (9.6%)	1 (2.27%)	4 (50%)	0.001	NA	NA	NA
Cardiogenic shock, *n* (%)	82 (3.8%)	57 (21.59%)	25 (12.76%)	0.014	NA	NA	NA
Timing, *n* (%)							
Pre coronary angiography	78 (95%)	57 (100%)	21 (84%)	0.007	NA	NA	NA
Post coronary angiography	4 (5%)	0	4 (16%)	0.007	NA	NA	NA
Coronary presentation, *n* (%)							
ST-elevation myocardial infarction	389 (18.2%)	214 (81.06%)	175 (89.29%)	0.016	NA	NA	NA
Non-ST-elevation acute coronary syndrome	1084 (50.7%)	50 (18.93%)	21 (10.71%)	0.034	578 (60.33%)	435 (60.41%)	0.868
Stable coronary artery disease	273 (12.77%)	NA	NA	NA	137 (14.32%)	136 (18.89%)	0.012
Silent ischemia/others	380 (17.78%)	NA	NA	NA	234 (24.45%)	146 (20.28%)	0.043
Left ventricular ejection fraction, mean (SD)	44.8 ± 11.6	40 (35–45)	50 (40–55)	< 0.001	45 (35–55)	50 (40–55)	< 0.001
LVEF ≤ 40%, *n* (%)	578 (27.0%)	98 (37.12%)	33 (16.84%)	< 0.001	291 (30.38%)	156 (21.67%)	< 0.001
LVEF 41–49%, *n* (%)	618 (28.91%)	101 (38.26%)	48 (24.49%)	0.002	286 (29.85%)	183 (25.42%)	0.045
LVEF ≥ 50%, *n* (%)	942 (44.1%)	65 (24.62%)	115 (58.67%)	< 0.001	381 (39.77%)	381 (52.92%)	< 0.001
Creatinine clearance (ml/min), mean (SD)	76.2 ± 24.2	90 (62–90)	83 (65–90)	0.016	90 (53–93)	83 (70–90)	0.002
Hemoglobin (g/L), median (IQR)	13.4 (12–14.6)	13.4 (12.2–14.6)	13 (11.5–14.5)	0.012	13.6 (12.3–14.9)	13.2 (11.7–14.3)	< 0.001

SD: standard deviation, CABG: coronary artery bypass graft surgery, PCI: percutaneous coronary intervention,, IQR: interquartile range, LVEF: left ventricular ejection fraction

**Table 3:** Comparison of the angiographic and procedural data between patients who had emergency left main (LM) vs. non-emergency LM revascularization

	Overall, *n* = 2138	Emergent LM revascularization n = 460 (21.5%)		*P*-Value	Non-emergent LM revascularization n = 1678 (78.5%)		*P* Value
		PCI (*n* = 264)	CABG (*n* = 196)		PCI (*n* = 958)	CABG (*n* = 720)	
Angiographic characteristics							
Medina classification, *n* (%)				< 0.001			< 0.001
1,1,1	760 (35.5%)	74 (28.03%)	62 (31.63%)	0.402	356 (37.16%)	268 (37.22%)	0.979
1,0,1	162 (7.6%)	11 (4.17%)	28 (14.29%)	< 0.001	37 (3.86%)	86 (11.94%)	< 0.001
0,1,1	393 (18.4%)	79 (29.92%)	45 (22.96%)	0.096	153 (15.97%)	116 (16.11%)	0.938
1,1,0	402 (18.8%)	60 (22.73%)	11 (5.61%)	< 0.001	250 (26.10%)	81 (11.25%)	< 0.001
1,0,0	202 (9.4%)	20 (7.58%)	23 (11.73%)	0.130	96 (10.02%)	81 (11.25%)	0.417
0,1,0	160 (7.5%)	11 (4.17%)	19 (9.69%)	0.016	45 (4.70%)	85 (11.81%)	< 0.001
0,0,1	59 (2.7%)	9 (3.41%)	8 (4.08%)	0.689	21 (2.19%)	21 (2.92%)	0.347
Lesion characteristics							
Isolated left main disease, *n* (%)	138 (6.5%)	22 (8.3%)	12 (6.1%)	< 0.001	81 (8.5%)	23 (3.2%)	< 0.001
LM + (triple-vessel disease), *n* (%)	1202 (56.2%)	98 (37.1%)	124 (63.3%)	< 0.001	481 (50.2%)	499 (69.3%)	< 0.001
LM + (LAD and LCx), *n* (%)	327 (15.3%)	50 (18.9%)	27 (13.8%)	< 0.001	138 (14.4%)	112 (15.6%)	< 0.001
LM + [RCA and (LAD or LCx)], *n* (%)	139 (6.5%)	20 (7.6%)	25 (12.8%)	< 0.001	44 (4.6%)	50 (6.9%)	< 0.001
LM + LAD, *n* (%)	287 (13.4%)	63 (23.9%)	8 (4.1%)	< 0.001	188 (19.6%)	28 (3.9%)	< 0.001
LM + LCx, *n* (%)	35 (1.6%)	10 (3.8%)	0 (0.0%)	< 0.001	20 (2.1%)	5 (0.7%)	< 0.001
LM + RCA, *n* (%)	10 (0.5%)	1 (0.4%)	0 (0.0%)	0.99	6 (0.6%)	3 (0.4%)	0.740
SYNTAX score				< 0.001			< 0.001
Low (≤ 22), *n* (%)	430 (20.2%)	81 (30.68%)	18 (9.18%)	< 0.001	223 (23.30%)	108 (15.17%)	< 0.001
Intermediate (23–32), *n* (%)	1107 (52.0%)	104 (39.39%)	83 (42.35%)	0.524	577 (60.29%)	343 (48.17%)	< 0.001
High (≥ 33), *n* (%)	592 (27.8%)	79 (29.92%)	95 (48.47%)	< 0.001	157 (16.41%)	261 (36.66%)	< 0.001
SYNTAX score, median (IQR)	28 (24–33)	28 (22–33)	32 (25–37)	< 0.001	28 (23–32)	29 (25–35)	< 0.001
Lesion location							
Ostial/shaft only, *n* (%)	536 (25.1%)	51 (19.32%)	59 (30.10%)	0.007	184 (19.21%)	242 (33.61%)	< 0.001
Distal bifurcation, *n* (%)	1602 (74.9%)	213 (80.68%)	137 (69.90%)	0.007	774 (80.79%)	478 (66.39%)	< 0.001
Procedure							
Intra-aortic ballon pump, *n* (%)	200 (9.4%)	62 (23.48%)	52 (26.53%)	0.454	36 (3.76%)	50 (6.94%)	0.003
Impella, *n* (%)	14 (0.7%)	6 (2.27%)	1 (0.51%)	0.247	6 (0.63%)	1 (0.14%)	0.250

CABG: coronary artery bypass graft surgery, PCI: percutaneous coronary intervention, LM: left main, LAD: left anterior descending, LCX: left circumflex, RCA: right coronary, SYNTAX: The SYNergy between percutaneous coronary intervention with TAXus and cardiac surgery, IQR: interquartile range

### Procedural technique

All PCIs were performed using second-generation DESs (drug-eluting stents). The most common type of stent used was the XIENCE family (Abbott Vascular- Santa Clara, CA, USA) of everolimus-eluting coronary stent systems (65%). The most common PCI approach in emergency LMCA revascularization patients was the planned 2-stent strategy (80%). The most frequently performed type of CABG in emergency LMCA revascularization was conventional CABG (on-pump), accounting for 85% of patients. The left internal mammary artery was used in 98%, double mammary arteries in 5%, and radial conduits in 1% of cases, whereas 99% of patients received venous grafts.

### Discharge and follow-up medications

In emergent LMCA revascularization, patients who underwent PCI were more frequently discharged on P2Y12, beta-blockers, statins, and ACEi/ARBs (angiotensin-converting enzyme inhibitors/angiotensin II receptor blockers). No difference was seen in either type of revascularization strategy concerning aspirin (ASA) on discharge (Table 4). A similar pattern was observed at follow-up for P2Y12 inhibitors and ACEi/ARBs. ASA was more common in CABG patients than PCI patients (*p* = 0.032). No difference was observed between PCI and CABG patients concerning beta-blockers or statin prescriptions at discharge (Table 4).

**Table 4:** Comparison of the medications during discharge and follow-up between patients who had emergency left main (LM) vs. non-emergency LM revascularization

	Overall, *n* = 2138	Emergent LM revascularization *n* = 460 (21.5%)		*P*-Value	Non-emergent LM revascularization n = 1678 (78.5%)		*P* Value
		PCI (*n* = 264)	CABG (*n* = 196)		PCI (*n* = 958)	CABG (*n* = 720)	
Discharge medications							
ASA, *n* (%)	2010 (99.10%)	222 (99.55%)	166 (97.65%)	0.095	946 (99.58%)	676 (98.69%)	0.045
P2Y12 inhibitors, *n* (%)	1833 (90.40%)	223 (100%)	129 (75.88%)	< 0.001	946 (99.58%)	535 (78.10%)	< 0.001
Beta blocker, *n* (%)	1914 (94.40%)	216 (96.86%)	146 (85.88%)	< 0.001	915 (96.32%)	637 (92.99%)	0.003
Statin, *n* (%)	1934 (95.40%)	214 (95.96%)	139 (81.76%)	< 0.001	947 (99.68%)	634 (92.55%)	< 0.001
ACE inhibitors or ARB, *n* (%)	1516 (74.80%)	202 (90.58%)	86 (50.59%)	< 0.001	847 (89.16%)	381 (55.62%)	< 0.001
Medications during follow-up							
ASA, *n* (%)	1713 (97.10%)	174 (96.67%)	135 (100%)	0.032	859 (97.50%)	545 (95.78%)	0.068
P2Y12 inhibitors, *n* (%)	1129 (64.00%)	140 (77.78%)	83 (61.48%)	0.002	638 (72.42%)	268 (47.10%)	< 0.001
Beta blocker, *n* (%)	1674 (94.80%)	171 (95.00%)	129 (95.56%)	0.819	838 (95.12%)	536 (94.20%)	0.443
Statin, *n* (%)	1727 (97.80%)	174 (96.67%)	130 (96.30%)	0.859	873 (99.09%)	550 (96.66%)	0.001
ACE inhibitors or ARB, *n* (%)	1332 (75.50%)	159 (88.33%)	71 (52.59%)	< 0.001	765 (86.83%)	337 (59.23%)	< 0.001

ASA: aspirin, ACE: angiotensin-converting enzyme, ARB: angiotensin II receptor blocker, CABG: coronary artery bypass grafting, PCI: percutaneous coronary intervention

### In-hospital events

Univariable analysis was used to compare hospital events between PCI and CABG in patients with emergent and non-emergent revascularization. The emergent LMCA revascularization group had higher cardiac mortality (59/460, 12.8%) and total mortality (66/460, 14.35%) without a significant difference between PCI and CABG patients. There was no significant difference between PCI and CABG patients concerning MACCEs. The non-emergent LMCA revascularization group had lower cardiac mortality (22/1678, 1.3%) and total mortality (39/1678, 2.3%). CABG patients reported a higher mortality rate than PCI patients. MACCE and noncardiac mortality were significantly higher in CABG patients than PCI patients. In both the emergent and non-emergent LMCA revascularization groups, hospital stay was significantly longer in CABG patients (Table 5).

**Table 5:** Comparison of the hospital and follow-up events between patients who had emergency left main (LM) and non-emergency LM revascularization

	Overall, *n* = 2138	Emergent LM revascularization *n* = 460 (21.5%)		*P*-Value	Non-emergent LM revascularization *n* = 1678 (78.5%)		*P* Value
		PCI (*n* = 264)	CABG (*n* = 196)		PCI (*n* = 958)	CABG (*n* = 720)	
In-hospital events							
Cardiac death, *n* (%)	81 (3.8%)	36 (13.64%)	23 (11.73%)	0.546	3 (0.31%)	19 (2.64%)	< 0.001
Non-cardiac death, *n* (%)	24 (1.1%)	4 (1.52%)	3 (1.53%)	> 0.99	3 (0.31%)	14 (1.94%)	0.001
Myocardial infarction, *n* (%)	73 (3.4%)	15 (5.68%)	20 (10.20%)	0.070	3 (0.31%)	35 (4.86%)	< 0.001
Target lesion revascularization, *n* (%)	11 (0.5%)	3 (1.14%)	1 (0.51%)	0.640	2 (0.21%)	5 (0.69%)	0.147
Target vessel revascularization, *n* (%)	15 (0.7%)	5 (1.89%)	0	0.075	2 (0.21%)	8 (1.11%)	0.023
Cerebrovascular events, *n* (%)	48 (2.2%)	12 (4.55%)	13 (6.63%)	0.329	7 (0.73%)	16 (2.22%)	0.009
MACCE, *n* (%)	125 (5.8%)	38 (14.39%)	28 (14.29%)	0.974	16 (1.67%)	43 (5.97%)	< 0.001
Congestive heart failure, *n* (%)	89 (4.2%)	17 (6.44%)	32 (16.33%)	0.001	32 (3.34%)	8 (1.11%)	0.003
Major bleeding, *n* (%)	313 (14.6%)	39 (14.77%)	34 (17.35%)	0.455	125 (13.06%)	115 (15.97%)	0.092
Minor bleeding, *n* (%)	157 (7.3%)	17 (6.44%)	18 (9.18%)	0.272	50 (5.22%)	72 (10%)	< 0.001
Total mortality, *n* (%)	105 (4.9%)	40 (15.15%)	26 (13.27%)	0.568	6 (0.63%)	33 (4.58%)	< 0.001
Duration of hospital stay, median (IQR) (Days)	7 (3–12)	5 (3–9)	13 (9–17)	< 0.001	3 (0–5)	11 (8–15)	< 0.001
Follow-up events							
Cardiac death, *n* (%)	14 (0.7%)	5 (2.59%)	2 (1.23%)	0.889	3 (0.33%)	4 (0.60%)	0.524
Non-cardiac death, *n* (%)	25 (1.3%)	2 (1.04%)	0	0.237	10 (1.09%)	13 (1.94%)	0.043
Myocardial infarction, *n* (%)	50 (2.6%)	3 (1.63%)	10 (6.29%)	0.005	21 (2.33%)	16 (2.41%)	0.665
Target lesion revascularization, *n* (%)	52 (2.72%)	8 (4.35%)	8 (5.03%)	0.371	27 (2.99%)	9 (1.35%)	0.141
Target vessel revascularization, *n* (%)	57 (2.98%)	4 (2.17%)	8 (5.03%)	0.046	27 (2.99%)	18 (2.71%)	0.936
Cerebrovascular events, *n* (%)	29 (1.64%)	4 (2.22%)	4 (2.96%)	0.636	11 (1.25%)	10 (1.76%)	0.465
MACCE, *n* (%)	108 (6.1%)	15 (8.33%)	16 (11.85%)	0.099	48 (5.45%)	29 (5.09%)	0.955
Congestive heart failure, *n* (%)	415 (19.4%)	83 (31.44%)	24 (12.24%)	0.008	212 (22.13%)	96 (13.33%)	< 0.001
Major bleeding, *n* (%)	7 (0.4%)	0	0		5 (0.57%)	2 (0.35%)	0.614
Minor bleeding, *n* (%)	31 (1.76%)	5 (2.78%)	4 (2.96%)	0.685	11 (1.25%)	11 (1.93%)	0.322
Total mortality, *n* (%)	39 (2.0%)	7 (3.63%)	2 (1.23%)	0.470	13 (1.41%)	17 (2.54%)	0.036
Median follow-up time (IQR) (months)	20 (10–37)	20 (13–31)	12 (3–33)	0.002	21 (12–39)	19 (9–37)	0.009

CABG: coronary artery bypass grafting, IQR: interquartile range, MACCE: major adverse cardiovascular and cerebrovascular events, PCI: percutaneous coronary intervention

### Follow-up events

**Fig. 1:**
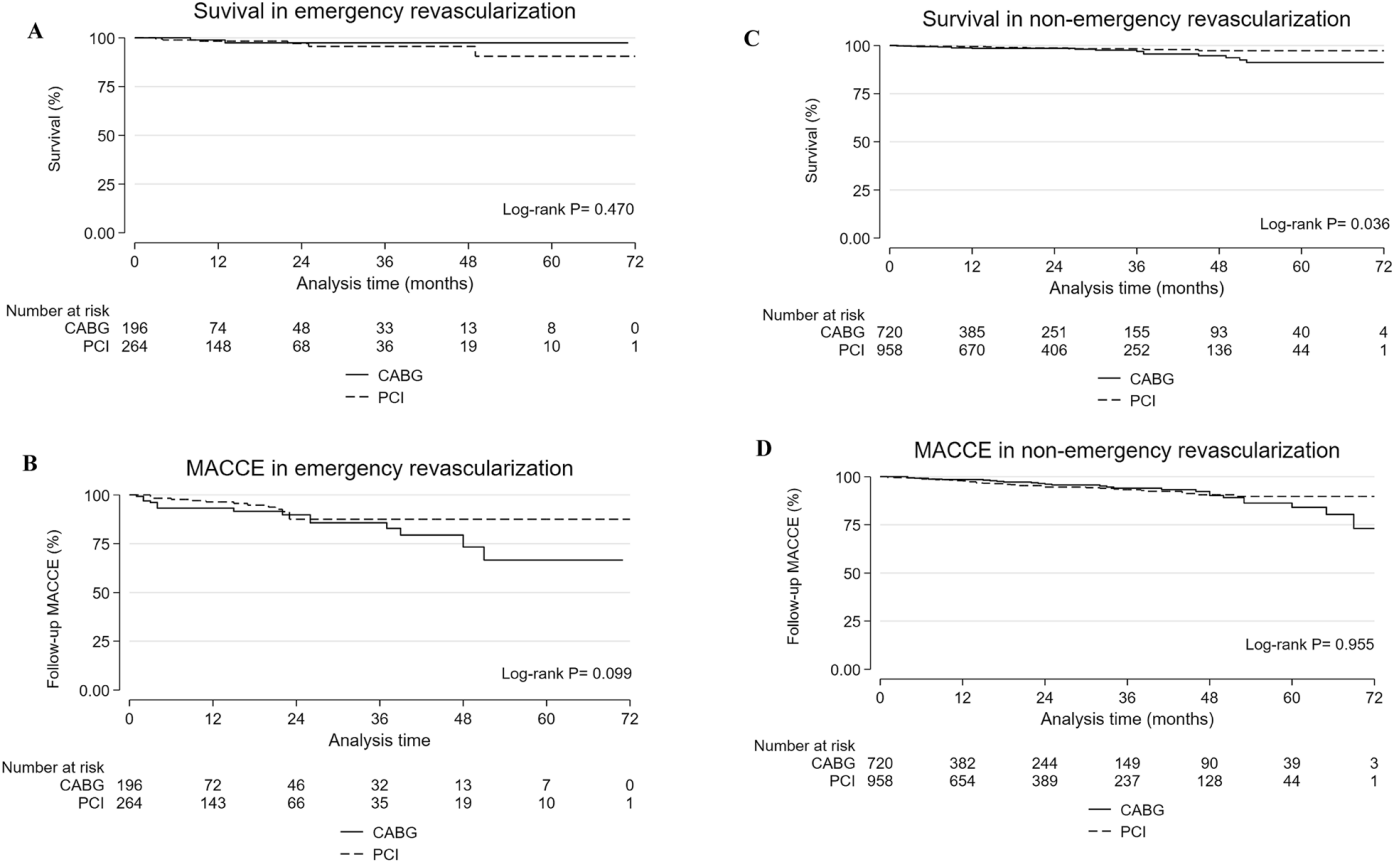
Total mortality and major adverse cardiovascular and cerebrovascular events (MACCE) in patients with emergent and non-emergent left main revascularization (PCI and CABG), at a median follow-up time of 20 months (IQR: 10-372). Total mortality **A** and **C**; MACCE **B** and **D**; percutaneous coronary intervention (PCI) (long dash); coronary artery bypass grafting (CABG) (solid line)

The median follow-up time was 20 months (IQR: 10–37 months). Those with emergent LMCA revascularization had a cardiac mortality of 7/460 (1.5%) and a total mortality of 9/460 (1.96%), without a significant difference between PCI and CABG. MACCEs were reported in 31/460 (6.74%) patients, with no significant difference between PCI and CABG. Follow-up myocardial infarction and target vessel revascularization were significantly higher in patients who underwent CABG than in those who underwent PCI. Congestive heart failure was significantly higher in PCI patients than in CABG patients. The non-emergent LMCA revascularization patients had a total mortality of 30/1678 (1.79%), noncardiac (1.37%), and cardiac (0.42%). The total mortality was significantly higher in CABG patients than in PCI patients. MACCE was reported in 77/1678 (4.6%) patients, with no significant difference between PCI and CABG (Table 5). Kaplan‒Meier curves showed no significant difference between CABG and PCI patients in emergent LMCA revascularization regarding all-cause mortality and MACCE (Figs. 1A, B). Kaplan‒Meier curves showed significantly higher all-cause mortality in non-emergent LMCA revascularization patients who underwent CABG, with no significant difference in MACCE between CABG and PCI (Figs. 1C, D).

### Multivariable analysis

Several models were constructed to adjust for risk factors for hospital MACCE and mortality in emergent and non-emergent LMCA revascularization. In patients with emergent LMCA revascularization, MACCE was significantly higher in patients with high EuroSCORE, whether they underwent PCI [OR: 5.13 (95% CI 1.98–13.34); *P* = 0.001] or CABG [OR: 4.35 (95% CI 1.51–12.57); P = 0.007] compared to patients with low EuroSCORE. However, there was no difference in MACCE according to the revascularization technique after adjusting for EuroSCORE [OR: 0.79 (95% CI 0.45–1.38); *P* = 0.409]. Different revascularization methods did not significantly affect MACCE in different SYNTAX score categories. In patients presenting with arrest, both PCI [7.64 (3.37–17.35); *P* < 0.001] and CABG [45.81 (11.29- 185.82); *P* < 0.001] were associated with significantly higher MACCE compared to other presentations. Additionally, PCI had significantly lower MACCE in patients with arrest than CABG [0.46 (0.24- 0.87); *P* = 0.017] (Table 6).

**Table 6:** Univariable and multivariable analysis for factors associated with hospital MACCE in emergency and non-emergency left main revascularization

Hospital MACCE	Univariable		Multivariable	
Emergency revascularization	OR (95% CI)	*P*	OR (95% CI)	*P*
Baseline characteristics				
Male	1.90 (1.05–3.44)	0.033	–	–
Age	1.02 (0.99–1.04)	0.111	–	–
Body mass index	0.99 (0.93–1.04)	0.642	–	–
Smoking	0.85 (0.49–1.47)	0.561	–	–
Diabetes mellitus	0.96 (0.54–1.69)	0.887	–	–
Dyslipidemia	1.02 (0.59–1.78)	0.936	–	–
Hypertension	1.89 (1.03–3.44)	0.038	–	–
Previous myocardial infarction	1.77 (1.04–3.01)	0.037	–	–
Chronic kidney disease	3.57 (2.04–6.24)	< 0.001	2.99 (1.62–5.50)	< 0.001
Peripheral arterial disease	3.22 (1.63–6.33)	0.001	2.45 (1.17–5.13)	0.017
Cerebrovascular accident	2.09 (0.97–4.50)	0.059	–	–
Congestive heart failure	3.88 (2.13–7.06)	< 0.001	2.67 (1.37–5.19)	0.004
Atrial fibrillation	1.92 (0.85–4.24)	0.109	–	–
Ejection fraction category	1.03 (0.74–1.41)	0.874	–	–
PCI vs. CABG	1.01 (0.60–1.71)	0.974	–	–
EuroSCORE				
EuroSCORE	1.08 (1.04–1.10)	< 0.001	2.69 (1.82–3.97)	< 0.001
PCI vs. CABG	1.01 (0.60–1.71)	0.974	0.79 (0.45–1.38)	0.409
Presentation				
Arrest	4.72 (2.63–8.50)	< 0.001	2.16 (1.11–4.22)	0.024
Shock	8.73 (4.87–15.64)	< 0.001	8.69 (4.47–16.92)	< 0.001
STEMI	0.26 (0.14–0.67)	< 0.001	–	–
NSTEMI	0.24 (0.13–0.44)	< 0.001	–	–
PCI vs. CABG	1.01 (0.60–1.71)	0.974	0.46 (0.24–0.87)	0.017
SYNTAX score				
SYNTAX score	1.02 (0.99–1.04)	0.158	1.38 (0.95–1.99)	0.091
PCI vs. CABG	1.01 (0.60–1.71)	0.974	1.14 (0.66–1.96)	0.641
Non–emergency revascularization				
Baseline characteristics				
Male	1.20 (0.66–2.19)	0.542	–	–
Age	1.01 (0.98–1.03)	0.553	–	–
Body mass index	0.97 (0.92–1.02)	0.232	–	–
Smoking	0.93 (0.55–1.59)	0.796	–	–
Diabetes mellitus	1.16 (0.65–2.05)	0.618	–	–
Dyslipidemia	2.79 (1.31–5.93)	0.008	2.36 (1.22–5.57)	0.013
Hypertension	1.68 (0.86–3.27)	0.127	–	–
Previous myocardial infarction	1.23 (0.71–2.15)	0.461	–	–
Chronic kidney disease	1.33 (0.73–2.42)	0.350	–	–
Peripheral arterial disease	1.01 (0.45–2.25)	0.989	–	–
Cerebrovascular accident	0.59 (0.21–1.65)	0.317	–	–
Congestive heart failure	1.59 (0.79–3.19)	0.192	2.36 (1.10–5.04)	0.027
Atrial fibrillation	–	–	–	–
Ejection fraction category	0.80 (0.59–1.08)	0.148	–	–
PCI vs. CABG	0.27 (0.15–0.48)	< 0.001	0.30 (0.16–0.56)	< 0.001
EuroSCORE				
EuroSCORE	1.06 (1.02–1.12)	0.005	1.07 (1.03–1.11)	0.001
PCI vs. CABG	0.27 (0.15–0.48)	< 0.001	0.25 (0.14–0.45)	< 0.001
Presentation				
Arrest	–	–	–	–
Shock	–	–	–	–
STEMI	–	–	–	–
NSTEMI	0.62 (0.35–1.11)	0.107	–	–
PCI vs. CABG	0.27 (0.15–0.48)	< 0.001	–	–
SYNTAX score				
SYNTAX score	1.02 (0.99–1.05)	0.117	1.17 (0.79–1.73)	0.444
PCI vs. CABG	0.27 (0.15–0.48)	< 0.001	0.27 (0.15–0.50)	< 0.001

MACCE: major adverse cardiovascular and cerebrovascular events, CABG: coronary artery bypass graft surgery, PCI: percutaneous coronary intervention, EuroSCORE score: European system for cardiac operative risk evaluation, STEMI: ST–segment elevation myocardial infarction; NSTEMI: non-ST-segment elevation myocardial infarction, OR: odds ratio, CI: confidence interval

In emergent LMCA revascularization, hospital all-cause mortality was significantly higher with PCI [36.78 (4.86–278.48); *P* < 0.001] and CABG [42.13 (5.35–331.75); *P* < 0.001] in patients with high EuroSCORE compared to low EuroSCORE. While there was no difference between both approaches after adjusting for EuroSCORE [0.87 (0.49–1.56); *P* = 0.647]. There was no interaction between PCI/CABG and SYNTAX score categories for their effect on hospital all-cause mortality. Mortality was significantly higher in patients presented with arrest and had CABG [133.09 (28.63- 618.50); *P* < 0.001] or PCI [4.81 (2.09–11.08); *P* < 0.001]; however, PCI was associated with lower mortality compared to CABG [0.43 (0.22–0.85); *P* 0.016] (Table 7).

**Table 7:** Univariable and multivariable analysis for factors associated with hospital mortality in emergency and non-emergency left main revascularization

Hospital mortality	Univariable		Multivariable	
Emergency revascularization	OR (95% CI)	*P*	OR (95% CI)	*P*
Baseline characteristics				
Male	1.90 (1.05–3.45)	0.033	–	–
Age	1.04 (1.01–1.06)	0.005	1.04 (1.01–1.07)	0.007
Body mass index	0.95 (0.89–1.01)	0.108	0.93 (0.87–0.99)	0.038
Smoking	0.56 (0.31–1.01)	0.052	–	–
Diabetes mellitus	0.88 (0.50–1.54)	0.665	–	–
Dyslipidemia	1.43 (0.80–2.55)	0.230	–	–
Hypertension	2.54 (1.34–4.82)	0.004	2.91 (1.38–6.16)	0.005
Previous myocardial infarction	1.52 (0.89–2.61)	0.127	–	–
Chronic kidney disease	1.81 (1.001–3.36)	0.05	–	–
Peripheral arterial disease	1.93 (0.93–4.01)	0.078	–	–
Cerebrovascular accident	2.09 (0.97–4.50)	0.059	–	–
Congestive heart failure	2.91 (1.58–5.38)	0.001	3.03 (1.50–6.14)	0.002
Atrial fibrillation	1.62 (071–3.69)	0.255	–	–
Ejection fraction category	1.43 (1.02–2.00)	0.036	2.68 (1.47–4.89)	0.001
PCI vs. CABG	1.17 (0.69–1.99)	0.568	–	–
EuroSCORE				
EuroSCORE	1.09 (1.06–1.13)	< 0.001	4.45 (2.83–7.01)	< 0.001
PCI vs. CABG	1.17 (0.69–1.99)	0.568	0.87 (0.49–1.56)	0.647
Presentation				
Arrest	10.57 (5.84–19.20)	< 0.001	5.40 (2.77–10.56)	< 0.001
Shock	11.53 (6.25–21.27)	< 0.001	9.07 (4.47 (18.42)	< 0.001
STEMI	0.20 (0.11–0.36)	< 0.001	–	–
NSTEMI	0.21 (0.12–0.39)	< 0.001	–	–
PCI vs. CABG	1.17 (0.69–1.99)	0.568	0.43 (0.22–0.85)	0.016
SYNTAX score				
SYNTAX score	1.01 (0.98–1.04)	0.044	1.36 (0.94–1.97)	0.099
PCI vs. CABG	1.17 (0.69–1.99)	0.568	1.31 (0.76–2.28)	0.331
Non–emergency revascularization				
Baseline characteristics				
Male	0.90 (0.411–1.98)	0.800	–	–
Age	1.02 (0.99–1.06)	0.168	1.04 (1.003–1.08)	0.031
Body mass index	0.96 (0.90–1.03)	0.287	–	–
Smoking	0.64 (0.32–1.28)	0.209	–	–
Diabetes mellitus	1.57 (0.74–3.33)	0.241	–	–
Dyslipidemia	3.88 (1.37–10.99)	0.011	3.56 (1.24–10.22)	0.018
Hypertension	1.79 (0.78–4.08)	0.167	–	–
Previous myocardial infarction	1.01 (0.50–2.05)	0.973	–	–
Chronic kidney disease	2.77 (1.45–5.30)	0.002	3.74 (1.83–7.62)	< 0.001
Peripheral arterial disease	1.37 (0.57–3.31)	0.485	–	–
Cerebrovascular accident	0.44 (0.11–1.84)	0.262	–	–
Congestive heart failure	2.01 (0.91–4.45)	0.083	–	–
Atrial fibrillation	0.57 (0.14–2.39)	0.441	–	–
Ejection fraction category	1.07 (0.72–1.57)	0.743	–	–
PCI vs. CABG	0.13 (0.05–0.32)	< 0.001	0.100 (0.04–0.25)	< 0.001
EuroSCORE				
EuroSCORE	1.12 (1.08–1.17)	< 0.001	1.13 (1.08–1.18)	< 0.001
PCI vs. CABG	0.13 (0.05–0.32)	< 0.001	0.11 (0.04–0.27)	< 0.001
Presentation				
Arrest	–	–	–	–
Shock	–	–	–	–
STEMI	–	–	–	–
NSTEMI	0.53 (0.26–1.10)	0.089	–	–
PCI vs. CABG	0.13 (0.05–0.32)	< 0.001	0.13 (0.05–0.32)	< 0.001
SYNTAX score				
SYNTAX score	1.05 (1.01–1.08)	0.005	1.38 (0.84–2.25)	0.201
PCI vs. CABG	0.13 (0.05–0.32)	< 0.001	0.14 (0.06–0.36)	< 0.001

CABG: coronary artery bypass graft surgery, PCI: percutaneous coronary intervention, EuroSCORE score: European system for cardiac operative risk evaluation, STEMI: ST–segment elevation myocardial infarction; NSTEMI: non-ST-segment elevation myocardial infarction, OR: odds ratio, CI: confidence interval

In non-emergent revascularization, PCI was associated with lower MACCE in patients with low [0.16 (0.04–0.70); *P* = 0.015] and intermediate [0.20 (0.09–0.48); *P* < 0.001] EuroSCORE compared to CABG in patients with the same EuroSCORE category. PCI was associated with lower MACCE in patients with low [0.09 (0.02–0.41); *P* = 0.002] and intermediate [0.30 (0.13–0.73); *P* = 0.008] SYNTAX scores compared to CABG with the same SYNTAX score category (Table 6).

In non-emergent revascularization, PCI was associated with reduced hospital mortality in patients with intermediate [0.12 (0.03–0.43); *P* = 0.001] and high [0.13 (0.03–0.47); *P* = 0.002] EuroSCORE compared to CABG with the same EuroSCORE category. PCI was associated with lower hospital mortality in patients with low [0.09 (0.01–0.89); *P* = 0.031] and intermediate [0.09 (0.02–0.39); *P* = 0.001] SYNTAX scores compared to CABG with the same SYNTAX score category (Table 7).

Multivariable Cox regression analysis revealed that factors that increased the follow-up MACCE were female sex, cardiac arrest, cardiogenic shock at presentation, low left ventricular ejection fraction, hypertension, and low body mass index. Emergent PCI had a lower rate of follow-up MACCE compared to CABG [HR: 0.30 (95% CI 0.14–0.66), *P* < 0.003], with no difference between elective CABG and PCI [HR: 0.79 (95% CI 0.47–1.31), *P* = 0.355]. Factors associated with follow-up mortality were age, hypertension, peripheral arterial disease, and high EuroSCORE II. There was no difference in follow-up mortality between emergency PCI and CABG [HR: 1.18 (95% CI 0.23- 6.08), *P* = 0.845]. However, elective PCI was associated with lower follow-up mortality than CABG [HR: 0.35 (95% CI 0.16–0.76), *P* = 0.008] (Table 8).

**Table 8:** Multivariable Cox regression analysis for factors affecting follow-up MACCE and all-cause mortality

	HR (95% CI)	*P*
Follow-up MACCE		
Female	2.47 (1.58–3.87)	< 0.001
Arrest	2.82 (1.06–7.49)	0.038
Ejection fraction	0.97 (0.95–0.98)	< 0.001
Hypertension	2.04 (1.16–3.58)	0.013
Body mass index	0.95 (0.91–0.99)	0.020
Group		
Elective PCI vs. CABG	0.79 (0.47–1.31)	0.355
Emergency PCI vs. CABG	0.30 (0.14–0.66)	0.003
Follow-up all-cause mortality		
Age	1.04 (1.004–1.07)	0.027
Hypertension	5.32 (1.26–22.45)	0.023
Peripheral arterial disease	2.91 (1.30–6.53)	0.010
EuroSCORE	1.07 (1.04–1.11)	< 0.001
Group		
Elective PCI vs. CABG	0.35 (0.16–0.76)	0.008
Emergency PCI vs. CABG	1.18 (0.23–6.08)	0.845

MACCE: major adverse cardiovascular and cerebrovascular events, CABG: coronary artery bypass graft surgery, PCI: percutaneous coronary intervention, EuroSCORE score: European system for cardiac operative risk evaluation, HR: hazard ratio

## Discussion

Several studies addressing revascularization of the LMCA disease focused on patients with chronic coronary artery disease; however, around 7% of patients with LMCA disease present with acute coronary syndrome [14]. Those patients are usually excluded from clinical studies; therefore, it is essential to evaluate the optimal revascularization strategy for LMCA disease in both elective and emergency situations [15]. The Gulf Left-Main Study investigated emergent LMCA revascularization and compared the outcomes of PCI versus CABG. The study found that PCI was associated with lower hospital MACCE and hospital all-cause mortality compared to CABG in patients presented with arrest or shock; however, there was no advantage of PCI vs. CABG in emergency revascularization if stratified by EuroSCORE or SYNTAX score. Additionally, PCI had lower follow-up MACCE compared to CABG in emergent LMCA revascularization within a 20-month follow-up period. PCI was more advantageous than CABG in non-emergency situations, especially in patients with low and intermediate EuroSCORE and SYNTAX scores. Despite being associated with high mortality, revascularization in the acute phase has been shown to improve prognosis [16].

The baseline characteristics differed between groups. The EuroSCORE II was significantly higher in patients with PCI compared to CABG in patients who had emergency revascularization, and there was a greater number of patients with left ventricular ejection fraction (LVEF) of less than 40% in the PCI group in emergency and non-emergency revascularization. However, EuroSCORE was higher in CABG patients with non-emergent revascularization than in PCI. Of patients who required LMCA revascularization, 21.5% were identified as needing revascularization emergently. The study also found that the incidence of LMCA being the culprit lesion in patients with acute myocardial infarction was 5%, which is consistent with a previous report [17, 18].

In a meta-analysis of 977 patients from 13 different studies, the thirty-day mortality was 15% post-emergent LMCA PCI, comparable to the data for the PCI group in this study [19]. In patients who required emergent LMCA revascularization using CABG, the estimated death rate was reported to be 19%, which is higher than the rate of 11.7% in this study, indicating a trend toward percutaneous revascularization for high-risk patients and considering surgical options only for lower-risk selected patients [20]. The choice of revascularization strategy is largely affected by the clinical status of the patients. Urgent revascularization with either PCI or CABG is essential in clinically unstable patients to improve survival [21]. Practically, PCI is more convenient in emergencies. We found that PCI had survival benefits and lower hospital and follow-up MACCE in patients with shock or cardiac arrest. The outcomes of emergency revascularization were affected by patients' characteristics rather than the revascularization technique, except in critically ill patients with either arrest or shock. Optimizing patients before PCI and the early use of mechanical circulatory support in those patients might improve the outcomes of emergency revascularization [22]. In this study, PCI was more advantageous for revascularizing non-emergent LMCA disease, especially in patients with low and intermediate risk stratification using EuroSCORE and SYNTAX scores.

Once discharged, the reported mortality in this study was low (1.23–2.59% CABG vs. PCI, respectively) compared to published reports of 10.5% mortality at one year with emergent PCI of LMCA lesions [23]. This study is the largest series to date to assess revascularization in patients requiring emergent LMCA intervention. This demonstrates that PCI was associated with better in-hospital and follow-up outcomes than CABG, especially in patients presented in critically ill conditions. This supports PCI as the intervention of choice for hemodynamically unstable patients in the setting of myocardial infarction. However, the study had some limitations, including its retrospective, nonrandomized design and the potential influence of unmeasured factors, such as the surgeons' experience and the volumes of the procedures at participating centers. Therefore, future randomized trials on this subset of patients are highly recommended to continue to define optimal revascularization strategies.

## Conclusions

Emergent LMCA revascularization is associated with high in-hospital mortality and morbidity. Nevertheless, patients who survive to discharge have a much better prognosis. PCI could be advantageous over CABG in revascularizing LMCA disease in emergencies. PCI could be preferred for revascularization of non-emergent LMCA in patients with intermediate EuroSCORE and low and intermediate SYNTAX scores.

## Data Availability

The datasets used and/or analyzed during the current study are available from the corresponding author on reasonable request.

## Abbreviations

LMCA: Left-main coronary artery
PCI: Percutaneous coronary intervention
CABG: Coronary artery bypass graft surgery
MACCE: Major adverse cardiovascular and cerebrovascular events
EuroSCORE score: European system for cardiac operative risk evaluation
CABG: Coronary artery bypass grafting
MACCE: Major adverse cardiovascular and cerebrovascular events
OR: Odds ratio
CI: Confidence interval
IQR: Interquartile range
HR: Hazard ratio
LV: Left ventricle
CAD: Coronary artery disease
EHR: Electronic health record
VIF: Variance inflation factor
EuroSCORE score: European system for cardiac operative risk evaluation
NSTE-ACS: Non-ST Elevation Acute Coronary Syndrome
SYNTAX: The SYNergy between percutaneous coronary intervention with TAXus and cardiac surgery
DES: Drug eluting stent
ACE: Angiotensin-converting enzyme
ARB: Angiotensin II receptor blocker
ASA: Aspirin
